# **­**A curated transcriptomic dataset collection relevant to embryonic development associated with *in vitro* fertilization in healthy individuals and patients with polycystic ovary syndrome

**DOI:** 10.12688/f1000research.10877.1

**Published:** 2017-02-23

**Authors:** Rafah Mackeh, Sabri Boughorbel, Damien Chaussabel, Tomoshige Kino

**Affiliations:** 1 Department of Human Genetics, Division of Translational Medicine, Sidra Medical and Research Center, Doha, 26999, Qatar; 2 Department of Systems Biology, Division of Translational Medicine, Sidra Medical and Research Center, Doha, 26999, Qatar

**Keywords:** Blastocysts, cumulus cells transcriptomics, embryos, Gene Expression Omnibus, granulosa cells, in vitro fertilization, oocytes, polycystic ovary syndrome

## Abstract

The collection of large-scale datasets available in public repositories is rapidly growing and providing opportunities to identify and fill gaps in different fields of biomedical research. However, users of these datasets should be able to selectively browse datasets related to their field of interest. Here we made available a collection of transcriptome datasets related to human follicular cells from normal individuals or patients with polycystic ovary syndrome, in the process of their development, during *in vitro* fertilization. After RNA-seq dataset exclusion and careful selection based on study description and sample information, 12 datasets, encompassing a total of 85 unique transcriptome profiles, were identified in NCBI Gene Expression Omnibus and uploaded to the Gene Expression Browser (GXB), a web application specifically designed for interactive query and visualization of integrated large-scale data. Once annotated in GXB, multiple sample grouping has been made in order to create rank lists to allow easy data interpretation and comparison. The GXB tool also allows the users to browse a single gene across multiple projects to evaluate its expression profiles in multiple biological systems/conditions in a web-based customized graphical views. The curated dataset is accessible at the following link: http://ivf.gxbsidra.org/dm3/landing.gsp.

## Introduction

Oocytes are maternal germ cells developed in ovaries during the fetal phase and kept throughout the female reproductive ages for monthly maturation and subsequent ovulation following the endocrinological regulation associated with menstrual cycles ^1^. Oocyte maturation starts with the monthly resumption of the first meiotic process of one primary oocyte arrested in prophase I (characterized by the germinal vesicle, also classified as immature or metaphase I (MI) stage) ^1^. After extrusion of the first polar body, the primary oocyte progresses to metaphase II of the second meiosis and becomes the secondary oocyte, which is competent to fertilization by a sperm.

Such oocyte growth/maturation occurs inside the ovarian follicle, which is also concomitantly under a process called folliculogenesis. Folliculogenesis consists of follicular cell proliferation, development and differentiation ^1^. Primordial follicles containing primary oocytes grow into the mature Graafian follicle with the coordinated progression of the holding germ cells to the secondary oocytes ^2^. Ovulation then occurs under the regulation of gonadotropins and sex steroids, resulting in the release of an oocyte into the peritoneal cavity. Upon fertilization by a sperm, the liberated oocyte resumes its second meiotic division to become the zygote, which further goes into a form of embryo called morula through several mitotic divisions and compaction of component cells. Continuous cell division further transforms morula to blastocyst, which has a fluid-filled cavity and is ready for implanting to the uterine endometrium ^3^.

The oocyte in the ovarian follicle is a primary regulator of follicular cell differentiation and function, whereas metabolic cooperation occurs between oocytes and follicular cells to ensure substrate supply necessary for oocyte growth/maturation ^4^. The follicular cells consist of two types of cell groups, theca cells (also known as stromal cells) and granulosa cells. Theca cells form the outer layer of the ovarian follicle, while inner granulosa cells make a direct contact with the oocyte. These cells also produce steroid hormones, such as progestins and estrogens, under the control of pituitary gonadotropins, which is important for priming uterine endometrium and other reproductive tissues for supporting expected implantation and pregnancy ^5^. During folliculogenesis, granulosa cells continuously proliferate to form the follicular antrum, a fluid-filled cavity formed among the granulosa cell cluster. Upon formation of the antrum, two populations of granulosa cells become identifiable: one cell group known as **cumulus cells** (CCs), which surround the oocyte and remain associated with it even after ovulation, and the other group called **mural granulosa cells**, which form an inner layer of the follicle. The oocyte and CCs form the cumulus-oocyte-complex in which these cells directly communicate with each other through the gap-junctions created between them. This cellular communication plays a central role in the regulation of folliculogenesis and oocyte maturation by enabling the nutritional transfer and traffic of macromolecules between them ^6^.

*In vitro* fertilization (IVF) is one type of assisted reproductive technology developed for the treatment of infertility ^7^. It is a procedure consisting of (1) harvesting oocytes from the peritoneal cavity of the women artificially stimulated for their ovulation, (2) fertilization of the oocytes by mixing with sperms *in vitro*, and (3) implantation of fertilized oocytes into the uterine cavity. Before implantation, fertilized oocytes are regularly cultured for 2–6 days in a growth medium allowing its cell division and multiplication. Although a lot of improvements have been added to IVF, its success rate for successful live birth is still less than 50% even in younger women, and the main challenge remains the risk of multiple pregnancies, which is directly associated with increased incidence of fetal morbidity and infant mortality during maternal, perinatal and neonatal periods ^8^. To prevent multiple IVF-associated pregnancies, single-embryo transfer is considered, for which selection of the most viable and healthy embryo is critical. Morphological inspection of embryos is employed for selecting high quality embryos ^9, 10^, but it is not sufficient to predict the developmental potential of embryos. Therefore, studies have been performed during the last several years to develop better methods of embryo selection by examining proteomics or metabolomics of embryos ^11– 13^. Recently, emergence of microarray technology has introduced a new approach to study the genetic aspects of fertility. Primarily, studies employing this new technique focused on the role surrounding follicular cells for evaluating the quality of carrying oocytes, and estimated its usefulness by comparing and correlating the data from stromal cells with the quality of embryos and with a positive or negative IVF outcome ^14– 19^. Such studies also included samples obtained from healthy or diseased women, for example women with polycystic ovary syndrome (PCOS), for whom the IVF success rate is known to be reduced compared with healthy subjects ^20^.

To help identify knowledge gaps in the field of IVF, ovarian function and/or the influence of reproductive diseases, we provide here a resource enabling mainstream researchers in this field to browse transcriptomic datasets relevant to the oocyte and surrounding stromal cells obtained from healthy subjects or those with PCOS, in association with IVF outcome. Such a resource offers a unique opportunity to identify the genes that play key roles in oocyte maturation, embryonic development and crosstalk between oocytes and granulosa cells, eventually contributing to the future improvement of the IVF procedure.

## Methods

In order to identify datasets relevant to IVF, we developed queries in a way to include the conditions, such as oocytes, CCs or granulosa cells in humans. Queries were employed on NCBI ( https://www.ncbi.nlm.nih.gov/) and are as follows:

- *Homo sapiens [organism] AND (oocyte OR oocytes) AND (“Expression profiling by array” [gdsType] OR “Expression profiling by high throughput sequencing” [gdsType])*.
- *Homo sapiens[organism] AND cumulus cells AND (“Expression profiling by array”[gdsType] OR “Expression profiling by high throughput sequencing”[gdsType])*.
- *Homo sapiens[organism] AND Granulosa cells AND (“Expression profiling by array”[gdsType] OR “Expression profiling by high throughput sequencing”[gdsType]).*
- *Homo sapiens[organism] AND (in vitro fertilization OR in vitro fertilization OR in vitro fecundation) AND (“Expression profiling by array”[gdsType] OR “Expression profiling by high throughput sequencing”[gdsType]).*

This query retrieved 85 datasets. After excluding RNA-seq datasets from the collection and examining each dataset carefully based on study description and list of samples and their annotations to verify their direct relevance to the theme of this data compendium, a total number of 23 datasets were selected. In total, 12 were successfully uploaded into the data browser. Details of these datasets are recapitulated in Table 1.

**Table 1.** Datasets* included in our collection.

GEO ID	Title	Platform	Number of samples	Genes expression used for dataset validation	Reference
GSE34526	Differential gene expression in granulose cells from polycystic ovary syndrome patients with and without insulin resistance: Identification of susceptibility gene sets through network analysis	Affymetrix	10	XIST FIGLA	27
GSE37277	Differentiating factors of cumulus cells related to quality of the human oocyte	Agilent 014850 v1	92	XIST	16
GSE37110	Differentiating factors of cumulus cells related to quality of the human oocyte	Agilent 014850 v1	32	XIST	16
GSE37117	Differentiating factors of cumulus cells related to quality of the human oocyte	Agilent 014850 v1	36	XIST	16
GSE37116	Differentiating factors of cumulus cells related to quality of the human oocyte	Agilent 014850 v1	24	XIST	16
GSE9526	Expression data from cumulus cells that surround oocytes resulting in early or late cleaving embryos	Affymetrix	16	XIST	17
GSE40400	Expression data from human cumulus cells isolated from oocytes at MI and MII staged in polycystic ovary syndrome (PCOS) patients	Affymetrix	8	XIST FIGLA	14
GSE10946	Gene expression microarray profiles of cumulus cells in lean and overweight-obese polycystic ovary syndrome patients	Affymetrix	23	XIST	15
GSE31681	Human cumulus cells	Affymetrix	24	XIST	28
GSE5850	Microarray analysis on NL and PCOS oocytes	Affymetrix	12	XIST	18
GSE43684	Modified natural and stimulated *in vitro* ferlitization cycle: Cumulus cells	Affymetrix	8	FIGLA	19
GSE12034	The transcriptome of human oocytes	Affymetrix	6	FIGLA, BMP15, XIST, Zp1, ZP2, ZP3	29

*: available at http://ivf.gxbsidra.org/dm3/geneBrowser/list.

After curation, each dataset was downloaded from the Gene Expression Omnibus of the National Center for Biotechnology Information website (NCBI GEO) using the SOFT file format, and was then uploaded, along with its study information and samples available, to the Gene Expression Browser, version 1.2 (GXB; http://ivf.gxbsidra.org/dm3/geneBrowser/list), an interactive web-based application developed at the Benaroya Research Institute (Seattle, WA, USA), hosted on the Amazon Web Services cloud ( https://github.com/BenaroyaResearch/gxbrowser) ( https://aws.amazon.com) ^21^. In GXB, we grouped the samples according to the expected future interpretation and comparison of study results. Each group contains samples of biological replicates, such as samples from control patients, and is compared to another group of samples. For example, Control group *vs* PCOS group, or Blastocysts group *vs* embryos of poor quality. Finally, computed ranking lists were created based on each grouping, using the rank list option provided in the GXB software. Therefore, GXB provides the users with a means to easily navigate and filter our uploaded and processed dataset collections, which are available at http://ivf.gxbsidra.org/dm3/landing.gsp.

A web tutorial for GXB is available online: http://ivf.gxbsidra.org/dm3/tutorials.gsp#gxbtut and is briefly reproduced here so that readers can use this article as a standalone resource ^21, 22^: “datasets of interest can be quickly identified either by filtering criteria from pre-defined lists shown on the left side of the GXB dataset navigation window, or by entering a query term in the search box located at its left top portion. Clicking on one of the studies listed in the dataset navigation window opens a viewer, which is designed to provide interactive browsing and graphic representations of the large-scale data in an interpretable format. This interface is intended to navigate ranked gene lists and displays transcriptomic results graphically in a context-rich environment. Selecting a gene from the rank-ordered list on the left side of the data-viewing window displays its expression values graphically. The drop-down menus directly above the graphical display give the users the following options: a) Change how the gene list is ranked, which allows the user to change the method used to rank the genes, or to include only the genes that are selected based on his/her specific biological interest; b) Change sample grouping (Group Set button), so that in some datasets, a user can switch between groups, based on, for example, the cell types and the diseases of interest; c) Sort individual samples within a group based on the associated categorical or continuous variables (e.g., gender and age); d) Toggle between the histogram and a box-plot plot with expression values, which are demonstrated as a single point for each sample in the graph; e) Paste color legends for sample groups; f) Select categorical information that is to be overlaid at the bottom of the graph. For example, the user can display gender or smoking status using this function; g) Provide a color legend for the categorical information overlaid at the bottom of the graph; h) Download the graph in a jpeg format. Generally, raw data of the measurements *per se* shown in graphs have no intrinsic utility in the absence of their contextual information. It is therefore important to display such information together with the data shown in the graphs, so that viewers are able to interpret demonstrated data and gain new insights from it. In the datasets provided, the contextual information has been organized under different tabs directly above the graphical display. The tabs can be hidden to make more room for displaying the data plots, or revealed by clicking on the blue “Show Info Panel” button in the top right corner of the display window. Information for the gene, which is selected from the list and is shown in the left side of the display, is available under the “Gene” tab. The study information is also available under the “Study” tab. Further, information on individual samples is provided under the “Sample” tab. Rolling the mouse cursor over a histogram bar while displaying the “Sample” tab enables viewing of any clinical, demographic, or laboratory information provided for the selected sample. Finally, the “Downloads” tab allows advanced users to retrieve the original datasets for their future analysis to be performed outside GXB. It also provides all available sample annotation data together with the expression data.” 

## Dataset validation

Quality checks for the datasets uploaded to GXB were performed by validating the specific expression of the Xist transcript (X-inactive specific transcript), which is a non-protein-coding RNA that inactivates one of the diploid X chromosomes existing in the female cells of mammals ^23, 24^. Since all uploaded datasets comprised samples obtained from women, Xist was expected to be present and expressed at high levels in all samples, except one dataset which comprises oocyte transcriptomic data, as haploid oocytes do not bear chromosome X inactivation. Expectedly, when microarrays provided probes for Xist, its expression was present in all datasets comprising cumulus or granulosa cells. While Xist expression was absent in oocyte samples of the GSE12034, it was highly expressed in the non-ovarian diploid tissue samples of the same dataset. Additional validation of our datasets was performed by examining the expression of some ovarian-specific genes, such as those specific to the zona pellucida protein (ZP1, ZP2 and ZP3), *FIGLA* (folliculogenesis-specific basic helix-loop-helix gene, also known as factor in the germline α), which encodes a transcription factor regulating the expression of multiple oocyte-specific genes ^25^, and *BMP15* (bone morphogenetic protein 15), which is functional in the folliculogenesis ^26^. *FIGLA* was selectively expressed in oocyte samples in the GSE12034 dataset, but not in non-ovarian control tissues. The same expression pattern was also confirmed for *ZP1*, *ZP2*, *ZP3*, and *BMP15*.

## Data availability

The data referenced by this article are under copyright with the following copyright statement: Copyright: © 2017 Mackeh R et al.

Data associated with the article are available under the terms of the Creative Commons Zero "No rights reserved" data waiver (CC0 1.0 Public domain dedication). 

All datasets were cited in our manuscript. They are designated by their GEO accession numbers (e.g. GSE34526), and can also be accessed using this identifier via the NCBI GEO website ( https://www.ncbi.nlm.nih.gov/gds/?term=). User can download all uploaded dataset files and associated sample information through the GXB tool: “Downloads” tab.
