# Supplementary material for: ­A curated transcriptomic dataset collection relevant to embryonic development associated with in vitro fertilization in healthy individuals and patients with polycystic ovary syndrome
Source: F1000Res. 2017 Feb 23;6:181. [Version 1] doi: 10.12688/f1000research.10877.1 (PMC5365227; doi:10.12688/f1000research.10877.1)
Supplement: Peer review history

# Peer review: Referee response for version 1

**DOI:** 10.5256/f1000research.11728.r20492

**Published:** 2017-03-20

**Authors:** Rita Singh

**Affiliations:** 1 Lab of Molecular Reproduction, Department of Zoology, University of Delhi, Delhi, India

The manuscript by Mackeh et al. is a collection of the gene expression datasets of oocyte, cumulus cells, and granulosa cells of normal and PCOS patients undergoing IVF. It is a good compilation of related datasets already published in public repositories.

 However, there are some concerns that are not addressed here.

 How were the expression values, as shown in the graphs, obtained from the raw data files? The details of the methodology used to analyse the raw data and to generate the ranked gene lists should be given. In the present form, it is difficult to make use of the data for any meaningful scientific analysis.

 The purpose of this study is to browse a single gene across multiple projects to evaluate its expression profiles in multiple biological systems/conditions in web-based customized graphical views. However, the gene expression data is shown as expression values for some datasets and as Log _2 _expression values for the others.

 There is a typographical mistake in the spellings of granulosa cells.

 The Pubmed articles linked to the data sets are not available.

 Although putting together these data is helpful for the analysis of transcriptome data from normal and PCOS patients undergoing IVF, it would be meaningful but not mandatory to include the data available from similar platforms for theca cells.

 It is a good effort done by the authors to put together several studies.

I have read this submission. I believe that I have an appropriate level of expertise to confirm that it is of an acceptable scientific standard, however I have significant reservations, as outlined above.

# Peer review: Referee response for version 1

**DOI:** 10.5256/f1000research.11728.r20579

**Published:** 2017-03-17

**Authors:** François J. Richard

**Affiliations:** 1 Département des Sciences Animales, Université Laval, Ville de Québec, QC, Canada

Although this manuscript is showing a web platform to look at genes involved in PCOS patients in oocyte, cumulus cells and granulosa cells, some concerns should also be considered and is not addressed here.

1. Bias can be obtained because of the method of RNA isolation, purification and RNA amplification that may be different between published papers.
2. The datasets were validated using cellular specific gene expression but nothing is mentioned about cellular contamination, reference genes (housekeeping genes), ... 

Because the data are classified by study using raw values for specific gene for each sample, it becomes highly difficult to grasp meaningful information. It would have been helpful to further analyze the data and not only showing the raw data of each sample.

 In conclusion, it seems to be a good platform to run a quick analysis looking at several studies.

I have read this submission. I believe that I have an appropriate level of expertise to confirm that it is of an acceptable scientific standard, however I have significant reservations, as outlined above.

# Peer review: Referee response for version 1

**DOI:** 10.5256/f1000research.11728.r20488

**Published:** 2017-03-01

**Authors:** Rawad Hodeify

**Affiliations:** 1 Department of Physiology and Biophysics, Weill Cornell Medical College in Qatar (WCM-Q), Doha, Qatar

The manuscript by Mackeh et al. presents a very interesting and novel approach to identify genes that are potentially linked to embryonic development. The authors introducing a valuable resource collecting gene expression profiling datasets from oocytes and surrounding stromal cells of healthy subjects or those with polycystic ovary syndrome, in correlation with IVF outcome. This resource is quite beneficial by providing a catalogue of genes that show altered expression in negative IVF outcome.

 The transcriptomic datasets are presented in an easy-to-use interactive web application that enables users, including those who are not experts in gene expression profiling, to identify altered gene expression in oocytes and associated cells in normal and diseased situations. Overall, I would give the manuscript in its current form a high priority to be indexed. I have minor comment and suggestion.

1. In page 3, the first paragraph (line 10) of the introduction, the secondary oocyte is also commonly known as ‘egg’. I would suggest that both terms are mentioned.
2. It will be interesting for the authors to check whether adding the term (Egg) in the queries will yield extra datasets.

I have read this submission. I believe that I have an appropriate level of expertise to confirm that it is of an acceptable scientific standard.

I have read this submission. I believe that I have an appropriate level of expertise to confirm that it is of an acceptable scientific standard.
